# Comparative Assessment of Reliability and Accuracy of Cone-Beam Computed Tomography (CBCT) Over Direct Surgical Measurement for Periodontal Bone Loss: A Prospective, Cross-Sectional Study

**DOI:** 10.7759/cureus.44608

**Published:** 2023-09-03

**Authors:** Anshuman B Patil, Nileshrao Patil, Romalpreet Singh, Priyanka Razdan, Sneha Singh, Rinnu A Mathew, Satyabrat Banerjee

**Affiliations:** 1 Department of Conservative Dentistry and Endodontics, Jawahar Medical Foundation's Annasaheb Chudaman Patil Memorial (JMF's ACPM) Dental College, Dhule, IND; 2 Department of Conservative Dentistry and Endodontics, Desh Bhagat Dental College, Malout, IND; 3 Department of Paediatric and Preventive Dentistry, Yogita Dental College and Hospital, Khed, IND; 4 Department of Conservative Dentistry and Endodontics, Rungta College of Dental Sciences, Bhilai, IND; 5 Department of Periodontics, Jawahar Medical Foundation's Annasaheb Chudaman Patil Memorial (JMF's ACPM) Dental College, Dhule, IND

**Keywords:** surgical measurements, alveolar bone loss, periodontal probing, cbct, probing depth

## Abstract

Introduction: Assessing bone condition holds significant value in the diagnosis, treatment planning, and prognosing the periodontal disease; its importance is undeniable. The main aim of the present study was to evaluate the accuracy of alveolar bone measurements due to periodontal disease using cone-beam computed tomography (CBCT), by comparing with surgical measurements, considered as the gold standard.

Materials and methods: A prospective cross-sectional study included a sample of 40 individuals diagnosed with chronic periodontitis who required periodontal surgery. A total of 202 sites were assessed for vertical and horizontal bone loss in the anterior (76 sites) and posterior (126 sites) teeth. Bone loss was measured using CBCT and a UNC 15 periodontal probe during the surgical intervention, and then compared. The statistical analysis involved employing a Student's t-test to compare measurements. Unpaired t-tests and correlation analyses were conducted using Pearson's correlation coefficient test. To establish statistical significance, a threshold of p<0.05 was considered appropriate.

Results: The statistical analysis carried out on the mean values of CBCT and direct surgical measurements for vertical bone loss demonstrated a significant difference (p<0.01). However, the values obtained for horizontal bone loss did not display statistical significance. A strong correlation of 0.94-0.99 existed between surgical and CBCT measurements. A statistically significant distinction was observed between the two methods in measuring bone loss at the distal and palatal sites of the anterior teeth.

Conclusion: Both CBCT and direct surgical measurement exhibit comparable accuracy potential in assessing alveolar bone loss. CBCT provides an accessibility advantage by enhancing visual access to challenging sites during surgical interventions, including palatal and distal areas of the teeth.

## Introduction

Over the past few decades, there has been a marked increase in the comprehension and interpretation of causation, pathogenesis, and management of inflammatory periodontal diseases. Nevertheless, the process of arriving at an accurate diagnosis and determining the most appropriate course of treatment continues to rely mainly on fundamental clinical techniques such as periodontal probing, which remains the most commonly used diagnostic tool for evaluating the health and attachment levels of periodontal tissues [1]. The influence of various factors, including probing force, amount of inflammation, type of periodontal probe used, tip diameter, tip angulation, and the location of probing, on the precision and reproducibility of clinical pocket probing depth (PD) is widely recognized. The presence of inaccuracies has the potential to greatly influence the process of clinical decision-making, especially in the context of continuous monitoring of periodontal status. Therefore, it is crucial to understand the potential sources of error when using periodontal probes to ensure accurate diagnosis and treatment planning [2].

Radiographs such as intraoral periapical radiographs (IOPAs), orthopantomograms (OPG), radiovisiography (RVG), and digital subtraction methods have been widely used to assess alveolar bone levels in periodontal defects [3]. All these procedures have inherent problems of lack of 3D information and difficulty in diagnosing hidden defects or furcation defects due to the overlapping of structures. Therefore, surgical exposure of the defect and measurements of bone levels came into being [4]. Although it provides useful and reliable information on bone levels, it also provides very little time for planning regenerative procedures.

In recent years, cone-beam computed tomography (CBCT) has emerged as a useful tool for diagnosis and treatment planning. It has low radiation exposure and is cost-effective compared to conventional computed tomography (CT) [5]. By presenting an image in 3D, CBCT can identify minute osseous defects related to periodontal diseases. Research findings demonstrate that CBCT possesses the same level of precision as direct assessments utilizing a periodontal probe and is just as dependable as IOPAs in identifying interproximal regions, which are currently presumed to have the highest resolution available [6,7].

Owing to limited studies on the accuracy of CBCT measurements in the assessment of periodontal defects compared with the gold standard method, the present study was conducted. The objective of this study was to assess the alveolar bone levels of teeth in chronic periodontitis, scheduled for periodontal surgery with CBCT, and to compare the measurements with periodontal probing during the surgical procedure, which is the gold standard of measuring alveolar bone loss.

## Materials and methods

This cross-sectional study was conducted in the Department of Periodontics, ACPM Dental College Dhule. Institutional ethical committee approval was sought (approval no-EC/NEW/INST/2022/2959/231). Written informed consent was obtained from all patients before the start of the study. This study was conducted in accordance with the principles of the Declaration of Helsinki.

Sample size calculation

In this study, GPOWER statistical software (ver. 3.1 Franz Faul, Universität Kiel, Kiel, Germany), considering a type 1α error of 0.05, power of 0.90, was used to evaluate the sample size [5]. The sample size was set to 35. Considering the sample attrition, we decided to increase the sample size to 40. The study was conducted on 40 patients (21 females and 19 males) with an average age range of 46.25±3.5 years.

Patient selection and eligibility

Sixty patients were screened for the presence of chronic periodontitis, and 40 patients were selected based on the following inclusion criteria: individuals who had been diagnosed with either chronic localized or generalized periodontitis affecting posterior teeth, with at least one site having a clinical PD of >5 mm and clinical attachment loss of >4 mm, who were above the age of 18, and who provided consent for flap surgery in at least one quadrant were eligible for the procedure. Smokers, pregnant or lactating females, patients with known medical conditions, patients who had undergone any type of periodontal surgery in the last six months, and patients who refused to provide their consent were excluded from the study.

Method

The patients were provided with routine oral prophylaxis procedures, such as oral hygiene instructions, scaling, and root planning using ultrasonic devices and hand instruments, before the surgical flap procedure.

CBCT measurements

**Figure 1:**
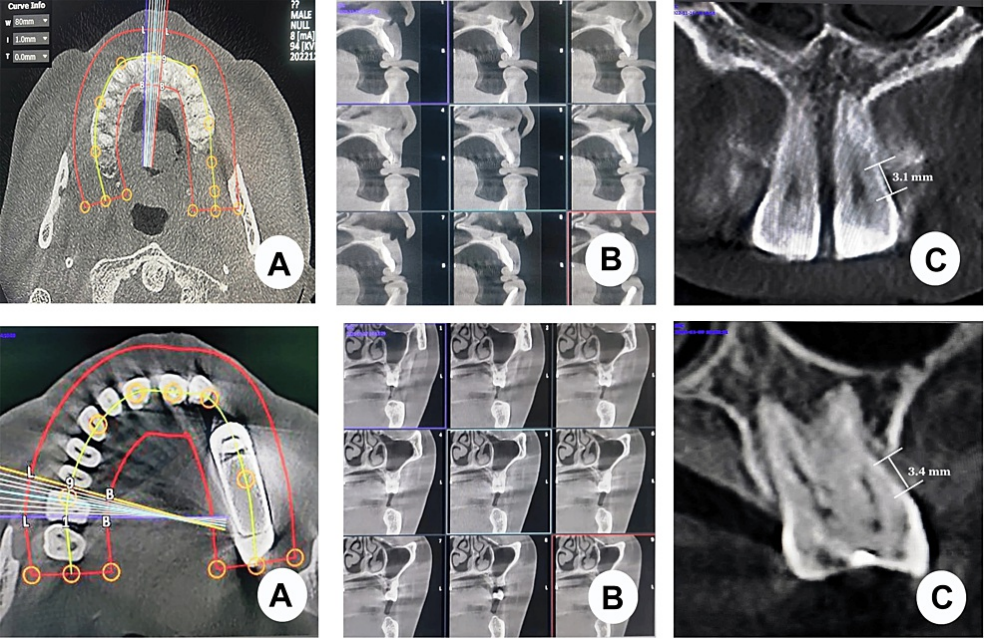
CBCT assessment of periodontal bone loss in anterior teeth (upper A, B, and C) and posterior teeth (lower A, B, and C). CBCT, cone-beam computed tomography

Prior to the surgical procedure, data from CBCT were obtained using a Planmeca Promax 3D machine. High-resolution images were acquired at a voltage of 90 kV, isotropic voxel size of 0.125 mm^3^, current of 10 mA, and exposure time of 13 sec (Figure 1).

Linear measurements were performed using in vivo software (Anatomage, San Jose, USA). The scans were viewed and analyzed in the coronal, sagittal, and cross-sectional planes. All measurements were recorded using the measurement tools in the software.

Clinical measurements of the periodontal defect

Following the meticulous aseptic protocol, the designated area was anesthetized and a complete thickness flap was lifted. After thorough debridement of the defects, hard tissue measurements of the alveolar bone loss at each site were performed according to Misch et al. for the anterior and posterior teeth [8].

For horizontal bone loss, the distance from the cementoenamel junction (CEJ) to the coronal part of the alveolar crest was measured and for vertical bone loss, the distance from CEJ to the coronal part of the alveolar crest was measured for the height of the defect, and from the CEJ to the base of the defect was measured for the depth of the defect. In the presence of restorations, instead of the CEJ, the apical part of the restoration was considered.

**Figure 2:**
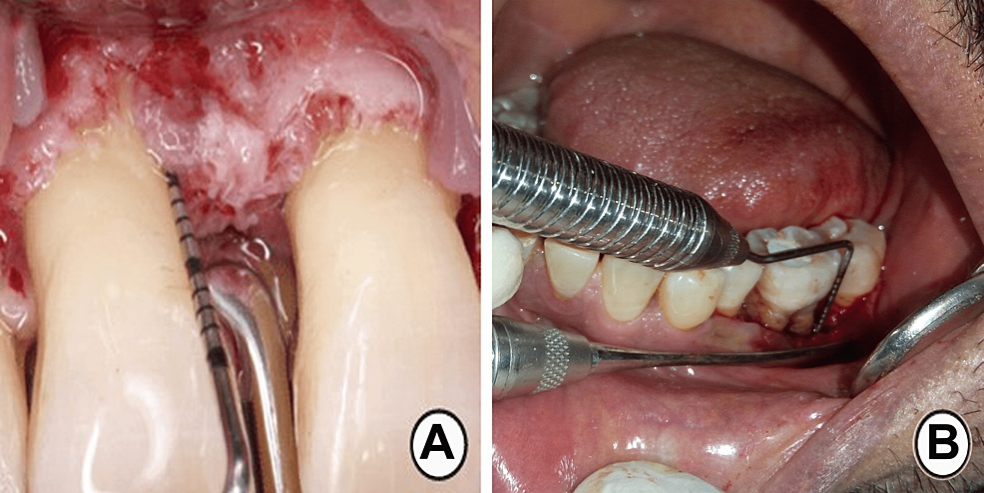
Direct surgical measurement of periodontal bone loss in (A) anterior teeth and (B) posterior teeth.

The measurements were performed using a UNC 15 periodontal probe calibrated in millimeters. (PCPUNC-15: HU-Friedy, Chicago, IL, USA). These measurements were performed on the buccal/labial, lingual/palatal, mesial, and distal aspects of the defects (Figure 2).

The measurements were rounded to the nearest millimeter. The same measurements were performed using CBCT and compared. After placement of the periodontal dressing, the flaps were sutured, and the patient was recalled after one week.

Reliability of the measurements

All clinical measurements were performed by a trained periodontist with 15 years of experience and CBCT measurements were performed by a trained radiologist with 18 years of experience. This was performed to reduce bias due to inter-examiner variability. Both clinicians were blinded to each other. A total of 202 defects were evaluated in 40 patients. The data obtained were subjected to statistical analyses.

Statistical analysis

The collected data, as a whole, were statistically analyzed using SPSS software version 22 (SPSS for Windows, Chicago, IL, USA). The Shapiro-Wilk test was used to check the normality of the data. Parametric tests were performed because the data were normally distributed. Student’s t-tests were used to compare the means of the CBCT measurements and direct surgical measurements. Pearson's correlation test was used to correlate the measurements between the two methods.

## Results

The mean age of the patients was 46.25±3.5 years. Males had more bone defects than females (110 defects in males and 92 defects in females). A total of 202 defects were assessed (76 defects in the anterior teeth, and 126 defects in the posterior teeth). Four sites were examined for anterior teeth (buccal, palatal, mesial, distal), and six sites were examined for posterior teeth (buccal, palatal, mesio-buccal, mesio-palatal, disto-buccal, disto-palatal),

The anterior teeth displayed more horizontal bone loss than did the posterior teeth, which had more vertical bone loss. There was no statistically significant difference in the measurements of horizontal bone loss in both the anterior and posterior teeth (p>0.05), whereas statistically significant differences were noted in the vertical bone loss measurements between the two methods (p<0.05), as shown in Table 1.

**Table 1:** Comparison of mean pocket depth of horizontal and vertical bone loss in anterior and posterior teeth by two study methods using Student’s T-test. *p-value <0.05: significant CBCT, cone-beam computed tomography

Type of bone loss	Region	Samples (n=202 sites)	Mean pocket depth in mm (CBCT)	Mean pocket depth in mm (Surgical)	p-value
Horizontal bone loss	Anterior teeth	52	3.21±1.67	3.54±1.09	0.768
	Posterior teeth	38	4.12±1.34	4.34±1.92	0.911
Vertical bone loss	Anterior teeth	24	4.15±1.34	4.34±1.98	0.001*
	Posterior teeth	88	5.98±1.09	6.23±1.56	0.004*

On comparison of different anatomical sites, non-significant differences were noted between CBCT and direct surgical measurements for all sites in the posterior teeth, whereas statistically significant differences were seen for distal and palatal sites of the anterior teeth (p<0.05), as shown in Table 2 and Table 3.

**Table 2:** Comparison of mean pocket depth in anterior teeth at different anatomical sites using Student’s T-test. *p-value <0.05: significant CBCT, cone-beam computed tomography

Site	Samples (n=76 sites)	Mean pocket depth in mm (CBCT)	Mean pocket depth in mm (Surgical)	p-value
Buccal	8	4.12±1.81	4.32±1.87	0.112
Distal	12	3.92±1.32	3.78±1.23	0.001*
Mesial	32	4.21±1.92	4.43±2.01	0.083
Palatal	24	3.62±1.54	4.01±1.67	0.016^*^

**Table 3:** Comparison of mean pocket depth in posterior teeth at different anatomical sites using Student’s T-test. *p value <0.05: significant CBCT, cone-beam computed tomography

Site	Samples (n=126 sites)	Mean pocket depth in mm (CBCT)	Mean pocket depth in mm (Surgical)	p-value
Buccal	12	5.12±1.63	5.23±2.12	0.654
Disto-buccal	22	4.32±1.56	4.67±1.98	0.568
Disto-palatal	26	4.65±1.96	4.97±1.65	0.121
Mesio-buccal	26	5.67±1.98	5.77±2.34	0.975
Mesio-palatal	22	6.10±1.76	6.34±2.09	0.031*
Palatal	18	5.67±1.01	5.78±1.72	0.887

There was a strong correlation between the CBCT measurements and the direct surgical measurement method (0.94-0.99) as shown in Table 4 and Table 5.

**Table 4:** Intra-class correlation between parameters involved in bone loss in anterior teeth using Pearson correlation test. *p-value <0.05: significant CBCT, cone-beam computed tomography

Site	Method	Intra-class correlation	p-value
Buccal	CBCT/Surgical	0.992	<0.05*
Distal	CBCT/Surgical	0.931	<0.05*
Mesial	CBCT/Surgical	0.892	<0.05*
Palatal	CBCT/Surgical	0.946	<0.05*

**Table 5:** Intra-class correlation between parameters involved in bone loss in posterior teeth using Pearson correlation test. *p-value <0.05: significant CBCT, cone-beam computed tomography

Site	Method		Intra-class correlation	p-value
Buccal	CBCT/Surgical		0.956	<0.05*
Disto-buccal	CBCT/Surgical		0.945	<0.05*
Disto-palatal	CBCT/Surgical		0.989	<0.05*
Mesio-buccal	CBCT/Surgical		0..992	<0.05*
Mesio-palatal	CBCT/Surgical		0.983	<0.05*
Palatal	CBCT/Surgical		0.956	<0.05*

## Discussion

Although periodontal probes are extensively used to assess the gingival and periodontal health of dental patients and are regarded as the benchmark for direct surgical measurements of periodontal defects, the technique is highly sensitive and influenced by various factors such as probe placement angulation, probe type, tip diameter, clinician expertise, probing force, defect type, and inflammation level. Moreover, direct surgical measurements provide inadequate time to devise regenerative procedures for patients [2].

The implementation of CBCT has gained immense popularity across diverse domains of dentistry, owing to its efficacy in facilitating the transition from 2D to 3D diagnostic and treatment planning [9]. According to previous research, CBCT has proven to be considerably more effective than digital radiography in detecting Grade I furcation involvement, fenestration, dehiscence, and three-wall defects. This suggests that CBCT may be a reliable diagnostic tool for identifying periodontal conditions. The aforementioned findings indicate that CBCT could play a crucial role in the diagnosis of the initial stages of periodontal defects. Conversely, a timely and precise diagnosis of periodontal disease is of immense significance for successful treatment planning and a favorable prognosis. Therefore, the use of CBCT for suspected periodontal defects has become increasingly important [10].

According to a systematic review by Choi et al., out of 13 studies comparing CBCT with clinical measurements, only four in vivo studies were performed, with different parameters, making comparison difficult [4]. Most studies have been conducted on simulated bone defects in human dry skulls [11,12]. Therefore, the present clinical trial was conducted on patients with chronic periodontitis who were scheduled for flap surgery, where direct surgical measurements of horizontal and vertical bone defects were compared with CBCT.

A total of 202 defects were identified (76 anterior and 126 posterior) in the 40 patients. The two groups were equal in terms of sex allocation. The present study revealed that males had more periodontal defects than females, which is in accordance with previous studies [3,4,10]. Potential sources of variability could be attributed to the collective influence of sex-specific genetic architecture and circulating levels of sex steroid hormones. In this regard, men are believed to be more susceptible than women, which could be attributed to the combined effects of these factors. Most of these defects are located in the posterior region.

The findings derived from our study suggest that the use of CBCT is more precise in detecting alveolar bone loss in the posterior region than in the anterior region. There was no statistically significant difference between CBCT and direct surgical measurements for all posterior sites, but significant differences were noted between the two methods for the distal and palatal areas of the anterior teeth. The dissimilarity observed in the diagnostic precision of CBCT between the anterior and posterior teeth is plausibly attributable to the distinctive morphological features of the periodontal bone in each region. In the anterior region, the lingual plates are considerably thinner and tapered toward the alveolar crest compared to the posterior teeth. Reduced thickness of the bone plate is associated with diminished image resolution, which consequently reduces the accuracy of linear measurements, particularly in the case of anterior teeth [13]. One explanation for this phenomenon is that distal site accessibility is challenging during periodontal surgeries as well as during instrumentation and measurement attempts using standard periodontal probing. Conversely, CBCT offers excellent accessibility for visualizing sites that are otherwise difficult to access during surgical intervention.

Additionally, our research revealed a strong correlation between direct surgical and CBCT measurements of bone defects. These results demonstrate that CBCT is a reliable and effective diagnostic tool for detecting bone defects. The present findings exhibit a high degree of agreement with the outcomes of all previous in vivo investigations [3,14]. Notably, the present study is one of the rare studies that has assessed and juxtaposed CBCT measurements of periodontal defects with actual surgical measurements in vivo. Therefore, the present study contributes to the extant literature by providing crucial insights into the comparability and reliability of CBCT measurements in periodontal defect assessment. Nonetheless, the current investigation did not align with Leung CC's research, which demonstrated poor precision of CBCT images in identifying bone defects because of restrictions in the spatial resolution of the CBCT equipment employed [15]. However, they conducted a study on human dry skulls, which could have led to the disparity in the results.

In the current investigation, in the measurement of horizontal bone loss, CBCT produced measurements almost similar to direct surgical measurements in measuring the distance from CEJ to the alveolar crest. However, in the case of vertical bone loss, a notable variance was noted between CBCT and direct surgical measurements with respect to the assessment of the depth of the defects compared to the height of the defects. The CBCT measurement methodology exhibited an underestimation of direct surgical measurements, which ranged from 0.2 to 0.4 mm. This might be due to the fact that CBCT was acquired before the surgical procedure. On the day of the surgical procedure, comprehensive removal of necrotic tissue from the bony abnormality may have led to the excision of certain bone fragments at the base of the abnormality, subsequently leading to more profound measurements of depth when compared to preoperative CBCT imaging. The alveolar crest, which is more cortical in nature, is less susceptible to bone removal during debridement. Consequently, measurements of the alveolar crest on CBCT may exhibit less deviation from direct surgical measurements. Moreover, owing to the more cancellous nature of the base of a bony defect, the probe may have penetrated marginally deeper during the procedure [1]. Similar findings have been reported by Grimard et al. [16]. Our findings contradict those of Misch et al. and Adurty et al., who reported non-significant differences in the height and depth of the defect [8,17]. Misch et al. conducted a study on human dry skulls, while Adurty et al. conducted research on a limited sample of 25 patients without specifying the number of defects analyzed, potentially contributing to the divergence of findings.

Radiographic examinations require proper justification and consideration of the ionizing radiation dose, including the use of 3D images. Although CBCT imaging is a useful tool, there are challenges concerning its image resolution, specifically voxel size. It should be noted that not all machines produce voxel sizes that can provide diagnostic value for the problem at hand. Moreover, the use of smaller voxel sizes often results in prolonged imaging exposure times and increased ionizing radiation exposure [18]. Therefore, it is crucial to carefully assess imaging parameters to ensure that the benefits of CBCT outweigh the risks associated with ionizing radiation. Appropriate justification and optimization of imaging procedures are essential to ensure that CBCT is safe and effective in clinical practice.

CBCT imaging is not the primary option for measuring periodontal bone defects. Rather, its use should be reserved for cases in which clinical and conventional images fail to provide adequate information for diagnosis and treatment decisions. It is imperative to select an appropriate field of view (FOV) size and exposure parameters to minimize the amount of radiation absorbed by the patient. This approach is in line with the ALARA principle, which advocates minimizing radiation exposure to a level as low as possible. Further, it is important to note that CBCT imaging should be carefully considered, and its benefits and drawbacks should be weighed before making a decision. Ultimately, the judicious use of CBCT imaging can enhance the accuracy and effectiveness of the diagnosis and treatment planning for periodontal bone defects.

Strengths of the study: As documented in the systematic review, the majority of the investigations were carried out on desiccated skulls that featured artificial bone impairments. Nevertheless, the present study was conducted in human subjects. Several investigations conducted on patients were found to have inadequate sample sizes. Our own research, on the other hand, involved a sample size of 40 individuals and assessed 202 bone defects, with a statistical power of 90%. We conducted an assessment of the height and depth of the defects, including the presence of vertical bone defects, which previous studies neglected to consider.

Limitations of the study

The main limitation of this study was its design. Being an observational study, it is subject to bias owing to a lack of randomization, which affects the validity of the study. The current research focused solely on assessing bone defects that were either horizontal or vertical in nature, without considering any furcation defects. Therefore, future randomized control trials are required to compare CBCT with other modalities for studying vertical, horizontal, and furcation defects.

## Conclusions

CBCT is a valuable tool that can complement clinical measurements and offer significant diagnostic information for evaluating bone defects. Moreover, the limited field of FOV associated with CBCT can produce high-resolution images with a reduced dose, comparable to 2D imaging. As a result, CBCT has gained popularity as an increasingly utilized imaging modality in dentistry. Considering radiation protection, it is imperative to demonstrate that the diagnostic information acquired through CBCT enhances treatment outcomes. The absence of such evidence would make this technique inappropriate for recommendation. This study centered on the diagnostic utility of CBCT in identifying periodontal lesions. If the study had emphasized treatment planning instead, CBCT could have been recommended as the preferred modality, offering distinct indications for resection procedures.
